# Identification of wood-boring beetles (Cerambycidae and Buprestidae) intercepted in trade-associated solid wood packaging material using DNA barcoding and morphology

**DOI:** 10.1038/srep40316

**Published:** 2017-01-16

**Authors:** Yunke Wu, Nevada F. Trepanowski, John J. Molongoski, Peter F. Reagel, Steven W. Lingafelter, Hannah Nadel, Scott W. Myers, Ann M. Ray

**Affiliations:** 1 Otis Laboratory, Center for Plant Health, Science and Technology, Animal and Plant Health Inspection Service, United States Department of Agriculture, Buzzards Bay, Massachusetts, United States of America; 2 Department of Ecology and Evolutionary Biology, Cornell University, Ithaca, New York, United States of America; 3 Animal and Plant Health Inspection Service, United States Department of Agriculture, Laredo, Texas, United States of America; 4 Animal and Plant Health Inspection Service, United States Department of Agriculture, Nogales, Arizona, United States of America; 5 Department of Biology, Xavier University, Cincinnati, Ohio, United States of America

## Abstract

Global trade facilitates the inadvertent movement of insect pests and subsequent establishment of populations outside their native ranges. Despite phytosanitary measures, nonnative insects arrive at United States (U.S.) ports of entry as larvae in solid wood packaging material (SWPM). Identification of wood-boring larval insects is important for pest risk analysis and management, but is difficult beyond family level due to highly conserved morphology. Therefore, we integrated DNA barcoding and rearing of larvae to identify wood-boring insects in SWPM. From 2012 to 2015, we obtained larvae of 338 longhorned beetles (Cerambycidae) and 38 metallic wood boring beetles (Buprestidae) intercepted in SWPM associated with imported products at six U.S. ports. We identified 265 specimens to species or genus using DNA barcodes. Ninety-three larvae were reared to adults and identified morphologically. No conflict was found between the two approaches, which together identified 275 cerambycids (23 genera) and 16 buprestids (4 genera). Our integrated approach confirmed novel DNA barcodes for seven species (10 specimens) of woodborers not in public databases. This study demonstrates the utility of DNA barcoding as a tool for regulatory agencies. We provide important documentation of potential beetle pests that may cross country borders through the SWPM pathway.

Global trade has created a pathway by which nonnative species may cross once impervious natural borders such as oceans and mountains^1^. Pathway is defined by the International Plant Protection Convention as a means that allows the entry or spread of a pest^2^. Global trade has been responsible for the introduction of some of the most damaging invasive species in the United States (U.S.), including Asian longhorned beetle (*Anoplophora glabripennis* [Motschulsky])^3^, citrus greening pathogen (*Candidatus* Liberibacter spp.)^4^, cogon grass (*Imperata cylindrica* [L.] P. Beauv.)^5^, and zebra mussels (*Dreissena polymorpha* [Pallas])^6^. The expansion of trade will likely increase opportunities for introduction and establishment of nonnative pest species^7^. Invasive animals, plants, and pathogens are a serious threat to all countries, as they alter and/or degrade the environment and may outcompete native species^5^. The establishment of invasive pest species imposes an economic burden on federal, state, and municipal governments that can escalate into billions of dollars annually^8^.

The economic impact of nonnative wood-boring insects is estimated to be among the greatest of invasive species in the U.S.^9^,^10^. The primary route of entry for wood-boring insects is as larvae infesting solid wood packaging material (SWPM) used to secure commodities (i.e., pallets, crates, and dunnage)^11^. The global movement of SWPM is regulated by the International Plant Protection Convention, International Standards for Phytosanitary Measures Rule No. 15 (ISPM 15)^2^, which requires SWPM to be treated to reduce the introduction of wood-boring insects. Current approved treatments are conventional and dielectric heat, and fumigation with methyl bromide. However, despite being marked with ISPM 15 compliance stamps, SWPM infested with live wood-boring larvae is still regularly intercepted by agriculture specialists from the U.S. Department of Homeland Security, Customs and Border Protection (CBP) at ports of entry in the U.S.^11^. Interceptions of nonnative species in commodities entering the U.S. are tracked by the United States Department of Agriculture (USDA) Pest Interception Database (Pest ID). Longhorned beetles (Cerambycidae) and metallic wood-boring beetles (Buprestidae) account for nearly 30 percent of interceptions associated with SWPM in the last three decades^11^, and typically these insects are intercepted as larvae^12^. However, due to highly conserved morphology, larvae are difficult to identify beyond family level^13^. Furthermore, larvae are often intercepted as early instars and may lack diagnostic characters associated with later instars^14^,^15^. When SWPM is found to be infested with the larvae of woodborers, cargo is re-exported and resources are rarely allocated to identify the immature insects found in the shipment^16^. Valuable regulatory and pathway risk information is lost when larvae remain unidentified; thus it is important to identify live species intercepted in SWPM to better characterize threats to forests, orchards, and urban landscapes.

Rapid identification of unknown specimens may be achieved through DNA barcoding and query of public sequence databases^17^. This approach has been adopted for use in regulatory fields such as conservation biology, consumer protection, and border biosecurity, especially when morphological identification of target taxa is difficult and/or impossible (e.g.,^18^,^19^,^20^,^21^). The ability of DNA barcoding to link immature life stages to adult beetles makes it a valuable tool for overcoming the challenge presented by conserved larval morphology^22^. However, the application of DNA barcoding depends on the availability of matching reference barcodes within public databases, where the order Coleoptera is still underrepresented compared with Hymenoptera, Diptera, and Lepidoptera^23^,^24^. The lack of coverage in Coleoptera can be mitigated by addition of new reference barcodes through morphological identification of adult voucher specimens, ultimately aiding in identification of any life stage in the future.

With this study, we sought to improve the capacity to identify nonnative wood-boring beetle larvae intercepted in SWPM by using a combination of DNA barcoding and rearing larvae to the adult stage. This integrated approach, which to our knowledge has not been used before, allows for cross validation of identification results. Novel DNA barcodes generated using this method are morphologically validated and will help bridge coverage gaps in public barcode databases. Data compiled in our study add to the record of woodborers inadvertently transported in SWPM into the U.S and likely reveal taxa being transported to other parts of the world. Application of the data may facilitate identification of risk factors and at-risk pathways, which will help plant protection agencies focus responses to threats posed by the movement of infested SWPM.

## Results

### Rearing and DNA barcoding

**Figure 1:**
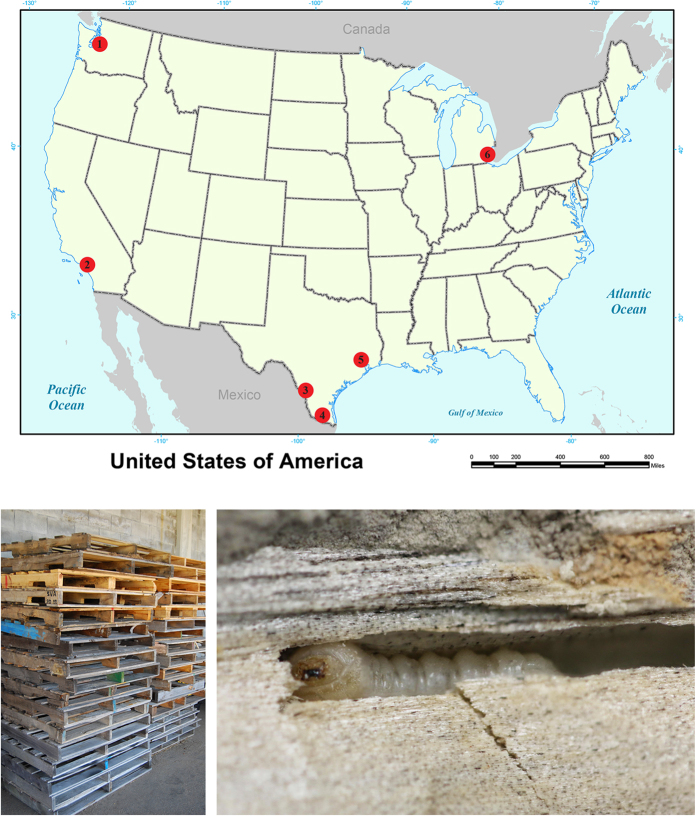
Participating U.S. ports of entry and wood packaging material. Upper: the ports are located at (1) Seattle, WA; (2) Long Beach, CA; (3) Laredo, TX; (4) Pharr, TX; (5) Houston, TX; (6) Romulus, MI. Lower left: an example of SWPM (pallets). Lower right: a cerambycid larva found inside SWPM (map prepared in ArcGIS [10.3.1] (www.esri.com) by: William Panagakos; photo credits: Peter Reagel & Kendra Vieira).

**Figure 2:**
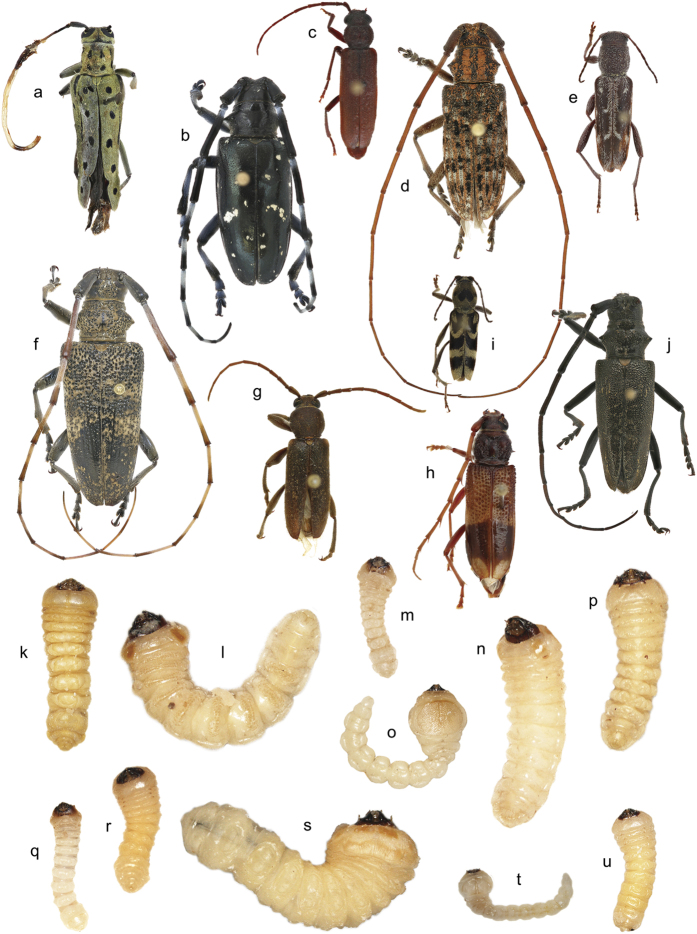
Examples of intercepted wood-boring larvae and reared adults from this study. The larvae depicted are visually similar and are difficult to identify below the family level. Cerambycidae: (**a**) *Saperda perforata*; (**b**) *Anoplophora glabripennis*; (**c**) *Arhopalus rusticus*; (**d**) *Monochamus alternatus*; (**e**) *Xylotrechus sagittatus*; (**f**) *Monochamus galloprovincialis*; (**g**) *Trichoferus campestris*; (**h**) *Phoracantha recurva*; (**i**) *Chlorophorus diadema*; (**j**) *Monochamus sartor*; (**k**) *Xystrocera globosa*; (**l**) *Acalolepta* sp.; (**m**) *Arphopalus rusticus*; (**n**) *Monochamus galloprovincialis*; (**p**) *Anoplophora glabripennis*; (**q**) *Arhopalus* sp.; (**r**) *Trichoferus* sp.; (**s**) inconclusive identification; (**u**) *Trichoferus campestris*. Buprestidae: (**o**) *Chrysobothris igniventris*; (**t**) *Buprestis* sp. Specimens are not shown to scale (photo credits: Sindhu Krishnankutty & Peter Reagel).

From April 2012 to March 2015, we received 387 immature insects found by CBP agriculture specialists in SWPM from six ports (Fig. 1). Most of the intercepted specimens were cerambycid larvae, whereas buprestid larvae were less commonly intercepted (9.8% of intercepted specimens; Fig. 2). Ninety-three larvae (24.0% of intercepted specimens) survived to the adult stage (Fig. 2), only two of which were buprestids. We obtained DNA barcode sequences for 358 of the 387 insects. No insertions/deletions or premature stop codons were found in the sequences, suggesting no amplification of nuclear mitochondrial DNA (NUMT). Barcode sequences could not be obtained for 29 specimens: three specimens failed to amplify in PCR reactions and 26 produced unreadable trace data despite multiple sequencing attempts (Supplementary Tables S1, S2).

Queries of the Barcode of Life Database v3 (BOLD)^25^ indicated that 312 of the 358 barcoded specimens belong to Cerambycidae and 35 specimens belong to Buprestidae. The number of cerambycids and buprestids identified to species or genus through barcoding alone was 265. Sixty-three cerambycids and 19 buprestids did not fall within 3% similarity to any sequences in BOLD, and thus could not be identified beyond the family level based on DNA barcode alone and therefore were considered inconclusive. Many specimens were identified only to genus level because: (1) no reference sequence existed in BOLD within 1% divergence of the query specimen; (2) the reference sequence that matched with our query was only identified to genus; and/or (3) the query sequence matched multiple congeneric taxa (e.g., most of our samples of *Anastrangalia* closely matched with both *A. dubia* [Scopoli] and *A. reyi* [Heyden], and thus were called *Anastrangalia* sp.). Identifications were confirmed by BLAST searches in GenBank. COI barcodes of 11 specimens did not group with either Cerambycidae or Buprestidae (Supplementary Table S2). Those specimens included woodwasps (Siricidae), ironclad beetles (Zopheridae), false darkling beetles (Melandryidae), and three parasitoid wasps (Braconidae, Ichneumonidae, and Aulacidae).

### Intercepted cerambycid specimens

Agricultural specialists submitted 338 cerambycids. DNA barcodes were obtained for 312 individuals, of which 249 specimens were identified to genus or species by querying sequences in BOLD; the remaining 63 were determined to be inconclusive beyond the family level. Morphological identifications were obtained for 91 individuals, including 65 adults identified by both barcoding and morphology. No conflicts were found between DNA barcoding and morphological identification in cases where both types of data existed. Morphology confirmed that 10 inconclusive sequences represented novel DNA barcodes from seven species not previously represented in BOLD, i.e., *Arhopalus syriacus* (Reitter), *Pseudastylopsis* sp. (closely related *P. squamosus* Chemsak & Linsley), *Pogonocherus perroudi* Mulsant, *Xylotrechus buqueti* (Castelnau & Gory), *X. magnicollis* (Fairmaire), *X. rufilius* Bates, and *X. smei* (Castelnau & Gory). The two approaches together yielded 275 cerambycids identified to genus or species.

**Figure 3:**
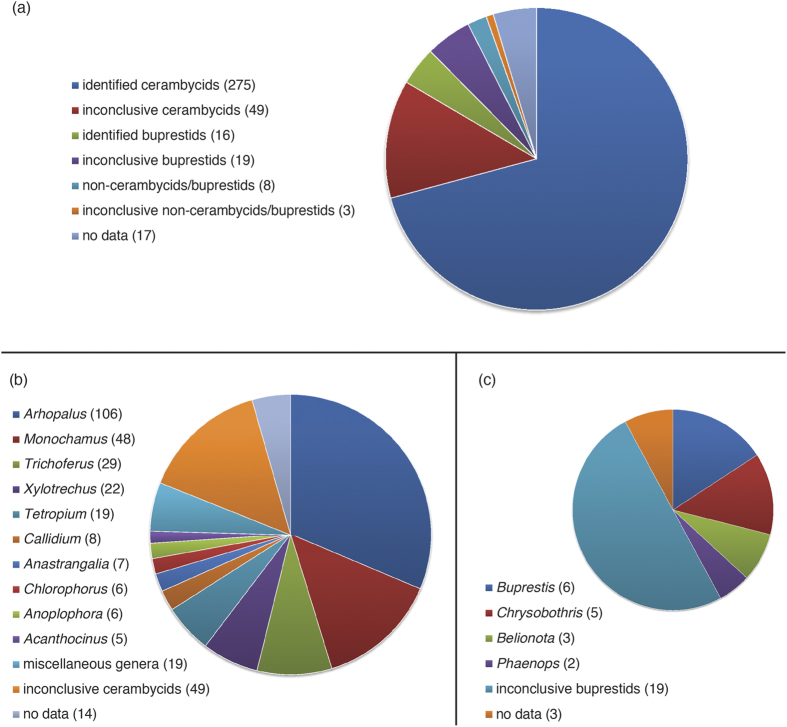
Proportional charts of wood-boring insects intercepted at the participating ports. Identification results reflect both morphology and DNA barcoding. (**a**) Summary of all samples; (**b**) summary of cerambycid genera; (**c**) summary of buprestid genera. Specimen counts are in parentheses. Miscellaneous cerambycid genera with less than five individuals can be found on Fig. 4.

The cerambycids identified in this study were members of 23 genera (Fig. 3), representing subfamilies Cerambycinae, Lamiinae, Lepturinae, and Spondylidinae. According to USDA more than half of the cerambycids identified during this study (149/275) were categorized as pests or potential pests. The five most frequently intercepted genera, *Arhopalus* (106 specimens), *Monochamus* (48 specimens), *Trichoferus* (29 specimens), *Xylotrechus* (22 specimens), and *Tetropium* (19 specimens), accounted for 66.0% of intercepted cerambycids. These five genera include important pests, including *Tetropium fuscum* (F.) and *Trichoferus campestris* (Faldermann). At least 25 individuals of the latter species were intercepted at multiple ports participating in the survey. Another highly destructive forest pest, the Asian longhorned beetle, was intercepted six times.

**Figure 4:**
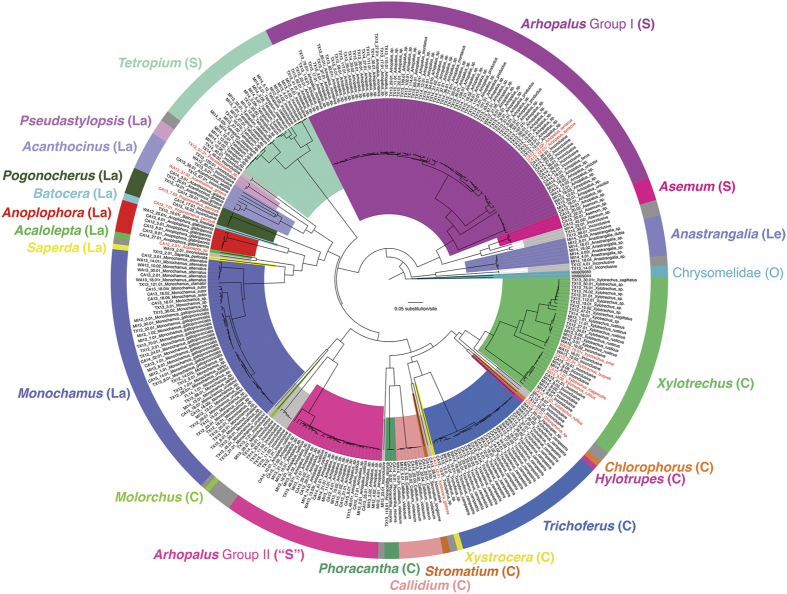
The Bayesian tree of intercepted cerambycids based on COI barcodes. Genera are displayed in different colors. Letters in parentheses denote subfamilies: Le: Lepturinae; S: Spondylidinae; La: Lamiinae; C: Cerambycinae. Novel DNA barcodes are highlighted in red. Inconclusive specimens are shown in grey. Note that the specimens of *Arhopalus* that were intercepted in the present study comprise two divergent groups, clustering with Spondylidinae and Cerambycinae. Chrysomelidae (*Chrysomela populi* and *Cryptocephalus distinguendus*) was used as the outgroup.

The Bayesian tree generated from COI barcode sequences (Fig. 4) showed that individual specimens identified through morphology and/or DNA barcoding grouped into their respective genera and subfamilies, although monophyly of the subfamilies was not well-supported based on our limited dataset (Fig. 4). Spondylidinae was polyphyletic due to (1) a close affinity between *Tetropium* and Lamiinae; and (2) the genus *Arhopalus* not recovered as monophyletic, instead separating into two groups, one of which (Group II) nested within Cerambycinae. Uncorrected *p*-distances between the two groups of *Arhopalus* ranged from 12.9% to 17.4%, exceeding distances between other congeneric species sampled in this study. Tracking the origin of SWPM associated with overseas shipments showed that *Arhopalus* Group I (*A. syriacus, A. ferus* [Mulsant], *A. unicolor* [Gahan], *A. montanus* [LeConte], and A. *productus* [LeConte] and closely related species) were found in shipments mostly from North America and Asia, and Group II (*A. rusticus* and allies) were found in shipments that originated in Europe (Supplementary Table S3).

**Figure 5:**
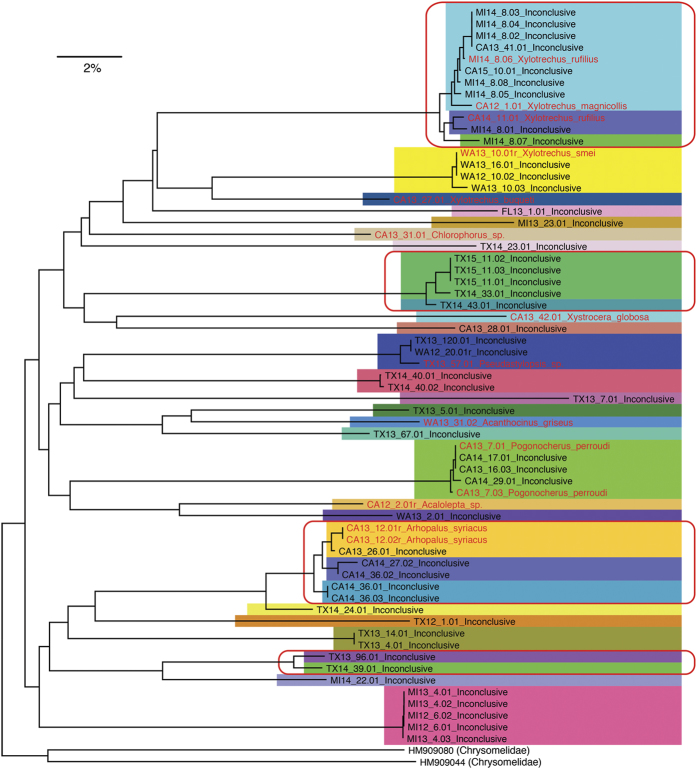
The neighbor-joining tree of cerambycid specimens lacking a close match in BOLD. Color blocks indicate specimens within 1% divergence from each other. Red rectangles cluster specimens that diverge less than 2% from each other. Specimens reared to adult are highlighted in red and represent novel DNA barcodes.

The 63 cerambycids not conclusively identified in BOLD (including 10 adults reared and later identified by morphology) were clustered into a neighbor-joining (NJ) tree (Fig. 5) to estimate the number of presumptive species among them based on genetic distance. Many individuals that did not survive to the adult stage clustered closely with morphologically identified specimens, suggesting conspecificity. By applying the 1% (BOLD threshold^25^) divergence threshold we assigned the specimens to 32 presumptive species; with the 2% (“barcode gap”^26^) divergence threshold the specimens were assigned to 26 presumptive species. The average uncorrected *p*-distance was 18.9% between individual specimens. The BIN Discordance Report implemented in BOLD confirmed the inference of 26 species by finding 11 concordant BINs and 15 singletons. Those singletons may represent species from taxonomic groups that are poorly-represented in BOLD.

### Intercepted buprestid specimens

**Figure 6:**
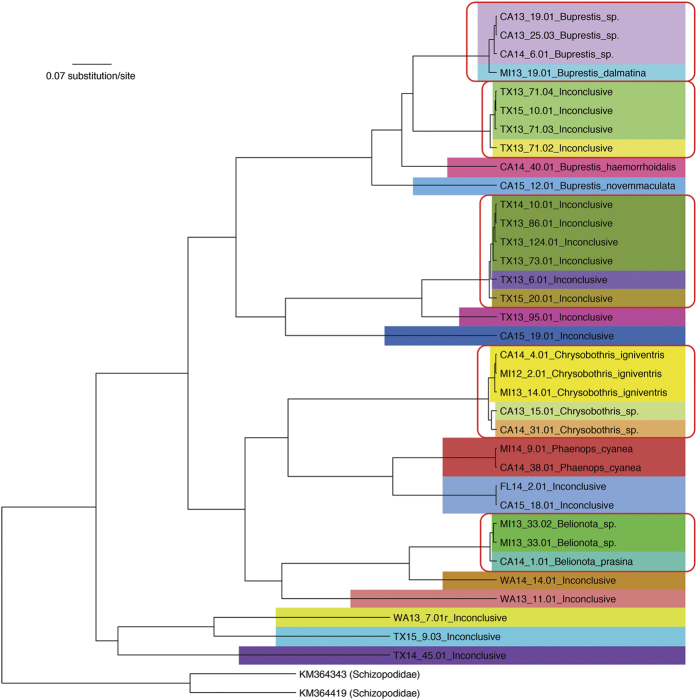
The Bayesian tree of intercepted buprestids based on COI barcodes. Color blocks indicate specimens within 1% divergence from each other. Red rectangles cluster specimens that diverge less than 2% from each other. Schizopodidae was used as the outgroup.

The number of buprestid larvae (38 specimens) intercepted in SWPM was considerably smaller than that of cerambycid larvae, and only two larvae survived to the adult stage. Those two individuals were identified as *Belionota prasina* (Thunberg) and *Phaenops cyanea* (F.). We generated 35 COI barcodes, only 16 of which (including the two adults) matched reference sequences in BOLD (Fig. 3). The four genera identified (*Belionota, Buprestis, Chrysobothris*, and *Phaenops:* subfamily Buprestinae) included 12 individuals categorized as pests in Pest ID. The Bayesian tree (Fig. 6) based on the entire buprestid sample set showed that most of the taxonomically inconclusive specimens did not group with identified individuals, suggesting high species diversity within the samples despite a small number of specimens. Application of the conservative and liberal distance threshold parameters clustered all specimens into 23 and 16 presumptive species, respectively. The uncorrected *p*-distance averaged 17.2% between specimens. The BIN Discordance Report found the same 16 presumptive species as the liberal threshold.

## Discussion

The difficulty associated with identifying wood-boring insects arriving at ports hinders risk assessment and mitigation responses for species that could damage forests, orchards, or urban landscapes if established outside their native ranges. Our three-year survey revealed that interceptions by CBP port personnel prevent entry of a large number of live pests in SWPM. The movement of live wood-boring pests into the U.S. was reduced after the U.S. implemented ISPM 15 in 2006^10^. However, our findings demonstrated that wood-boring beetles at high risk of becoming invasive continue to arrive at U.S. borders in SWPM. Less than 2% of incoming international cargo is inspected at U.S. ports^27^, leaving a high possibility that potential pests may pass undetected. Noncompliance with ISPM 15 standards and treatment failures may be factors responsible for the continued movement of pests across borders^11^. Lack of compliance increases the risk of pest introduction for all countries participating in global trade.

To improve the capacity of regulatory personnel to identify cerambycids and buprestids intercepted in SWPM, we integrated DNA barcoding and larval rearing, an approach that, to our knowledge, has not been utilized in similar types of studies. The two approaches together yielded species- or genus-level identification for 275 cerambycid and 16 buprestid larvae (and occasionally pupae). In cases where both types of data were available, identification results were either identical or congruent. Considering the difficulty of rearing larvae and the lack of diagnostic morphological characteristics, DNA barcoding was substantially more successful than morphology alone (265 vs. 93). However, 10 novel DNA barcodes representing seven species were generated from reared adults, which will allow for identification of these species in the future without requiring a prolonged rearing process (one year on average). Overall, the proportion of specimens we identified to species (151 of 376: 40.2%) is much higher than previous studies that relied exclusively on morphology (e.g., less than 9% cerambycids and buprestids identified to species level^12^).

Our work and the resulting reference list for cerambycids and buprestids intercepted in SWPM enhance the capacity of CBP and USDA to identify intercepted wood-boring larvae. This study provides important documentation of high-risk pest species with potential to cross country borders through the SWPM pathway. Our list of intercepted beetles includes 23 genera of cerambycids and four genera of buprestids. Additional genera are likely to be present among the inconclusive specimens. In cases where specimens bear only genus-level identification (e.g., groups of *Arhopalus* sp.), the actual number of species may vary depending on the operational criterion of the species concept^28^, which could include fixed genetic distance thresholds for species boundary or phylogenetic reciprocal monophyly (see^29^ and references therein). Nevertheless, our data represent the most accurate and updated species/genus-level list of wood-boring cerambycids and buprestids associated with SWPM entering the U.S. Given relatively high identification success, we were able to establish many previously unknown adult-larval associations that may prove helpful to morphological and taxonomic studies and ultimately to the development of identification keys. We expanded the barcode database by generating new reference barcodes for seven reared cerambycid species, which will allow for identification of those species in future interceptions. Additionally, we identified parasitoid wasp species in some wood-boring larvae; the hosts could not be identified because the specimens were fully parasitized and thus the host DNA concentration was too low to be amplified by PCR. Parasitoid wasp associations may prove beneficial in the search for biological control agents. *Wroughtonia dentator* (F). and *Rhimphoctona* spp. are parasitoids of *Tetropium* and *Monochamus* larvae^30^,^31^, the most commonly intercepted cerambycid genera in SWPM (based on Pest ID records). Data from this study may also inspire taxonomic revisions of groups for which few or no molecular data were previously available, such as the genus *Arhopalus*, which, based on our analysis, is not monophyletic.

Despite success in applying DNA barcoding to identification of potential pests, we encountered several problems as end-users of public databases. Insufficient taxonomic coverage in BOLD and GenBank is the largest hurdle. Together, BOLD and GenBank represent 15% of the known diversity of arthropods but are heavily biased toward taxa involved in specific barcoding campaigns^32^. More than 20% of our DNA sequences had no close match, a problem noted by earlier authors^23^. Even though gaps in the databases are constantly being filled by new barcodes, such as those generated in this study, the question remains whether the pace of barcode accumulation can meet the growing demand for utilizing DNA barcoding for identification of specimens^32^. Another impediment is misidentification of voucher specimens that bear reference barcodes, which causes confusion for end-users and limits the utility of DNA barcoding. For example, the best match of a cerambycid larva (MI13_10.02) in BOLD was a specimen under the name *Tetropium castaneum* (L.) (99.4% identical to the query specimens). However, the next 21 sequence matches comprised 20 *Arhopalus rusticus* (L.) (97.4% sequence similarity) and one *A. syriacus*. (98.6% sequence similarity). The unidentified larva most likely belongs to *A. rusticus*; the other two references in BOLD were possibly misidentified as *T. castaneum* and *A. syriacus*. In other cases, such discrepancies cannot be readily resolved. For example, a cerambycid specimen (CA13_24.01) matched a sequence from a single *Arhopalus unicolor* at 99.4% as well as two *Cephalallus oberthuri* Sharp at 98.8%, while the following matches dropped to 88.8% and below. Assigning either species name to the specimen would have been arbitrary. Unfortunately, discrepancies such as the above examples will appear repeatedly when unidentified specimens are queried, until the misidentified record is corrected by the submitter. Additional problems in the databases are synonymy (e.g., *Trichoferus campestris* and *Hesperophanes campestris* [Faldermann]) and misspellings (e.g., *Monochamus urussovi* [Fischer von Waldheim] misspelled as *M. urussovii*), which can cause confusion for those who are unfamiliar with the taxa. Therefore, rigorous effort in generating, justifying, and curating records in public repositories is crucial for the effective application of DNA barcoding^17^. Continued efforts in barcoding and morphological identification will fill gaps in barcode coverage of certain taxonomic groups as well as reveal errors that must be corrected in public databases, ultimately producing a more complete and comprehensive identification tool.

Our data confirm that the threat posed by nonnative larvae transported in SWPM remains high^11^, as more than half of the 275 cerambycids and 16 buprestids identified during our survey are categorized as pests by USDA. Interestingly, emerald ash borer (*Agrilus planipennis* Fairmaire), an invasive buprestid killing millions of ash trees in North America and Europe, was not intercepted during our study, possibly because species of this genus feed almost entirely in the cambial region under the bark^12^, which has been stripped away to comply with a 2009 debarking amendment to ISPM 15^2^. However, emerald ash borer overwinters and pupates in the outer sapwood and therefore may still have the potential to enter the country undetected in SWPM^33^. Eight nonnative cerambycid species have established and become pests in North America since the 1980s^34^. Individuals of four of the eight cerambycid species were identified among our interceptions, indicating that larvae continue to arrive in SWPM and could contribute to new infestations. The Asian longhorned beetle, which has the potential to cause economic loss estimated as great as 669 billion dollars^35^, was intercepted six times during the study. Those interceptions occurred in all three years we surveyed, despite increased emphasis by regulatory agencies in the past decade to implement phytosanitary treatment of SWPM. Another pest with potential to damage forest and fruit trees is *Trichoferus campestris*, detected in Quebec, Canada and 11 U.S. states, including Utah^34^,^36^. Our survey identified 25 specimens of *T. campestris* distributed among 15 separate interceptions. Two additional cerambycid pests established in North America are *Tetropium fuscum* and *Phoracantha recurva* Newman, each intercepted twice and comprising four and three larvae, respectively. *Monochamus urussovi*, a potential pest of pine, has not been previously identified as an intercepted pest in the U.S.^37^, although six larvae were intercepted in our study.

A potential application of identification of wood-boring pests in SWPM is the ability to link intercepted larvae to cargo destinations where pests were detected or became established. For example, among the 25 *Trichoferus campestris* that were intercepted, the intended destinations of some cargo packed with infested wood were locations in Salt Lake and Davis Counties in Utah. Populations of *T. campestris* have been detected in those counties and adjacent counties^37^. Our findings confirm that recurring movement of goods between domestic and non-compliant foreign trade partners may lead to repeated introductions, an outcome predicted by the model described by Colunga-Garcia *et al*.^38^. Repeated introductions may augment genetic diversity in invasive populations and increase colonizing success^39^,^40^. Our findings also highlight the necessity to identify commodities or wood treatment facilities associated with repeated introductions. Destinations with suitable climate and host conditions may be at an increased risk for establishment if invasive species are not intercepted at the ports. The study also determined that cargo with SWPM infested with Asian longhorned beetles were destined for Michigan, California, and Texas. Michigan is at a particularly high risk for establishment of new populations of Asian longhorned beetle, given the colonizing success of this species in nearby areas such as Illinois, Ohio and Toronto, in contrast with the western U.S., which generally lack suitable host tree species^41^. Trade partners associated with repeated introductions are not likely exclusive to the U.S. and thus other countries may also be at risk. Therefore, using DNA barcoding and morphology to link identities of intercepted invasive species with data on trade routes can inform regulatory agencies about areas at high risk of arrival or establishment.

## Conclusions

We identified 275 cerambycids and 16 buprestids intercepted in SWPM to genus or species by DNA barcoding and rearing larvae to the identifiable adult stage. We generated 265 reference DNA barcodes for the two beetle families, including 10 novel barcodes from seven cerambycid species not previously represented in public databases. The DNA barcodes obtained will aid in rapid and accurate identification of those potential pest species in future interceptions. Additionally, rearing larvae to adults may continue to add new reference barcodes to public databases. Despite errors and a lack of complete taxonomic coverage in public databases, DNA barcoding can be used for identification of nonnative insects crossing country borders. We documented that high-risk pest species such as Asian longhorned beetle and *Trichoferus campestris* were frequently intercepted. As a result of the increased volume of global trade, we can expect wood-boring insects associated with SWPM to continue to arrive at ports of entry worldwide. The ability to link potential pest species to trade partners and cargo destinations could provide the opportunity to monitor and assess the risk of repeated introduction through the same pathway. The integration of DNA barcoding and rearing presented in this study could serve as a valuable approach for identifying immature insects beyond the family level for biosecurity monitoring and phytosanitary regulation.

## Methods

### Materials

We partnered with six U.S. ports with the greatest number of intercepted cerambycids and buprestids during years 2007–2011 (based on Pest ID records). The participating ports were Long Beach, CA, Houston, TX, Laredo, TX, Pharr, TX, Detroit/Romulus, MI, and Seattle, WA (Fig. 1), four of which were also monitored by Haack and Petrice^42^. Additionally, four interceptions were received from the port of Miami, FL. From April 2012 to March 2015, CBP agriculture specialists at the participating ports collected live cerambycid and buprestid larvae (or occasionally pupae) discovered during inspection of SWPM. All samples were packed in coolers and shipped on ice to the USDA Otis Laboratory (Buzzards Bay, MA, U.S.) under USDA, Animal and Plant Health Inspection Service, Plant Protection and Quarantine 526 permit. When possible, the infested portion of the SWPM was shipped intact to provide natural host material for rearing larvae to the adult stage. If larvae were removed from or found outside the SWPM, they were placed in individual rearing cups with artificial diet developed for Asian longhorned beetle (modified from^43^). Early in the project, we attempted to rear buprestids on a diet developed for emerald ash borer^44^, but this was abandoned due to low survival. Sections of SWPM suspected or known to contain wood-boring larvae were placed in pouches made from aluminum window screen and held at 23 °C (ambient lab conditions) in covered barrels. Humidity was augmented in barrels weekly by misting wood samples with water using a hand sprayer. Larvae on diet were held in an environmental chamber at 25 °C and 65% relative humidity. Specimens were examined weekly for pupation and adult emergence. Larvae that arrived dead or died during the rearing process were stored in 95% ethanol at −20 °C prior to molecular analysis. Successfully reared adult insects were identified by personnel at the USDA Systematic Entomology Lab (Beltsville, MD, U.S.). Voucher specimens are retained in the reference collection at the Otis Laboratory. One or two legs and an antenna were removed from the right side of each adult specimen for DNA extraction.

### DNA extraction, amplification and sequencing

Genomic DNA was extracted using a DNeasy Blood and Tissue Kit (QIAGEN, Germantown, MD, U.S.) either from a segment of the leg or antenna (adults or pupae), from the muscular tissue inside the head capsule and mandibles (large larvae), or the whole voucher specimen (small larvae less than 3 mm). The primer pair LCO1490 and HCO2198^45^ amplified the standard 658 bp invertebrate barcode near the 5′ end of the mitochondrial cytochrome c oxidase I (COI). Amplification was conducted in a PCR reaction mix (20 μl total volume) containing 9 μl molecular grade water, 2 μl 10X PCR buffer without MgCl_2_, 2.8 μl MgCl_2_ (25 mM), 3.2 μl dNTP solution (1.25 mM), 0.4 μl primers (10 pmol/μl), and 0.2 μl of JumpStart Taq DNA Polymerase (Sigma Aldrich, St. Louis, MO) (2.5 units/μl). Two microliters of DNA were used as the template. Cycling conditions consisted of initial denaturation at 94 °C for 2 min, followed by 40 cycles of denaturation at 94 °C for 15 s, annealing at 52 °C for 30 s, extension at 72 °C for 1 min, and a final extension at 72 °C for 5 min. Negative controls were performed to monitor contamination. Amplified PCR products were examined on 3% agarose gels and subsequently purified by ExoSAP-IT (Affymetrix, Santa Clara, CA, U.S.) following the manufacturer’s protocol. Purified samples collected during the first year of the survey were sequenced on an ABI 3730XL DNA analyzer at University of Chicago Comprehensive Cancer Center DNA Sequencing and Genotyping Facility (Chicago, IL, U.S.). After the first year, samples were sequenced on an ABI 3730XL DNA analyzer at DNA Resource Core of Dana-Farber/Harvard Cancer Center (Cambridge, MA, U.S.). Sequences were manually edited in GENEIOUS 5.6.6 and subsequently aligned by the implemented MUSCLE algorithm. Uncorrected *p*-distances between sequences were calculated in GENEIOUS. Sequences were deposited in GenBank under the accessions KY357522–KY357879.

### Data analysis

All COI sequences were translated into amino acids to detect any insertions/deletions or premature stop codons, which would have indicated amplification of NUMT, a known problem in DNA barcoding of beetles^46^. To present the identified genera and species in the context of their genetic groupings, a phylogenetic tree based on the COI sequences was constructed using the Bayesian inference method in BEAST v1.8.2^47^ for each of the targeted beetle families. Two species of Chrysomelidae (*Chrysomela populi* L. and *Cryptocephalus distinguendus* Schneider) were chosen as the outgroup taxa for Cerambycidae, given the sister relationship between the two families^48^. We chose two species of Schizopodidae (*Schizopus laetus* LeConte and *S. sallei* Horn) as the outgroup for Buprestidae because their sister relationship was confirmed by Evans *et al*.^49^. The nucleotide substitution model GTR + I + G was selected for both taxonomic groups based on the Akaike Information Criterion obtained from JMODELTEST v2.1.4^50^. The species-tree prior assumed a Yule process. A Bayesian Markov chain Monte Carlo (MCMC) was run for 30 million generations sampled every 3000 generations. Chain convergence was assessed by effective sample size (ESS) values of the parameters in TRACER v1.5^51^. The first 25% of trees were discarded as burn-in.

Each sequence was submitted to BOLD v3 (as of 16 March 2015) as a query for species identification. If the query sequence showed less than 1% divergence to a reference barcode in BOLD, the species identification was confirmed; if the divergence was greater than 1% but smaller than 3%, or the query sequence matched with multiple taxa (presumably congeneric), the specimen was assigned to the same genus as the reference^25^. When a genus-level match was not obtained (i.e., divergence between the query and reference exceeded 3%), we considered the identification as inconclusive. Identification results were further confirmed by BLAST searches in GenBank’s nucleotide database.

In an effort to estimate the number of presumptive species (also called operational taxonomic unit^52^) present among specimens with inconclusive identification, we employed two analyses. First, we used fixed distance thresholds generally considered empirical criteria for a species boundary. We used both the 1% divergence (conservative threshold), where BOLD provides a species identification with a high degree of confidence^25^, and the 2% divergence (liberal threshold), which is considered a “barcode gap” (the genetic divergence among species in the COI region of the mitochondrial genome) observed in many animal taxa^28^. To graphically summarize the results, a NJ tree was constructed under the Kimura 2-parameter substitution model in MEGA v5.20^53^,^17^. Clusters on the NJ tree formed by sequences within 1% or 2% uncorrected *p*-distances were considered separate species. Given that only 38 buprestids were received from participating ports, we did not construct a NJ tree for inconclusive buprestid sequences, which were included with identified specimens on the Bayesian tree. The second analysis, performed to validate the number of presumptive species among the unmatched sequences, used the BIN Discordance Report^52^ implemented in BOLD Workbench. This method was more complicated than simple distance thresholds because it grouped sequences into clusters (i.e., presumptive species) through single linkage clustering and Markov refinement^52^. Each sequence was submitted to BOLD and received a BIN assignment.

For larvae that were reared to adults, we unified the results from DNA barcoding and morphology by assigning the identity based on the method that provided the lowest level of classification (i.e., species level). For example when DNA barcoding identified a specimen as *Monochamus sutor* and morphological examination only obtained genus level identification (*Monochamus* sp.), the sample was determined to be *Monochamus sutor*. We regarded the two results as congruent because the genus-level identification matched and the species identifications were not in conflict. We are confident in the unification of the results from the two approaches because both are reliable means of identification^18^,^19^,^20^,^21^,^25^. DNA barcoding results were determined with an emphasis towards avoiding over-diagnosis by only assigning genus level identification to the specimen when it matched with multiple species in BOLD with less than 1% divergence^25^.

## Additional Information

**How to cite this article**: Wu, Y. *et al*. Identification of wood-boring beetles (Cerambycidae and Buprestidae) intercepted in trade-associated solid wood packaging material using DNA barcoding and morphology. *Sci. Rep.* **7**, 40316; doi: 10.1038/srep40316 (2017).

**Publisher's note:** Springer Nature remains neutral with regard to jurisdictional claims in published maps and institutional affiliations.

## Supplementary Material

Supplementary Tables
