# Supplementary material for: Identification of wood-boring beetles (Cerambycidae and Buprestidae) intercepted in trade-associated solid wood packaging material using DNA barcoding and morphology
Source: Sci Rep. 2017 Jan 16;7:40316. doi: 10.1038/srep40316 (PMC5238391; doi:10.1038/srep40316)
Supplement: Supplementary Tables [file srep40316-s1.pdf]

Identification of wood-boring beetles (Cerambycidae and Buprestidae) intercepted in trade-associated solid wood packaging material using DNA barcoding and morphology

Yunke Wu[1,2]*, Nevada F. Trepanowski[1], John J. Molongoski[1], Peter F. Reagel[1,3], Steven W. Lingafelter[4] , Hannah Nadel[1], Scott W. Myers[1], Ann M. Ray[5]

[1] Otis Laboratory, Center for Plant Health, Science and Technology, Animal and Plant Health Inspection Service, United States Department of Agriculture, Buzzards Bay, Massachusetts, United States of America

[2] Department of Ecology and Evolutionary Biology, Cornell University, Ithaca, New York, United States of America

[3] Animal and Plant Health Inspection Service, United States Department of Agriculture, Laredo, Texas, United States of America

[4] Animal and Plant Health Inspection Service, United States Department of Agriculture, Nogales, Arizona, United States of America

[5] Department of Biology, Xavier University, Cincinnati, Ohio, United States of America

Supplementary information:

Table S1. Sample ID, GenBank accession number, and identification result for intercepted cerambycids and buprestids.

Table S2. Sample ID, GenBank accession number, and BOLD results for submitted insects identified as neither Cerambycidae nor Buprestidae.

Table S3. Origin of overseas shipments and associated SWPM for two groups identified as *Arhopalus* by morphology and/or DNA barcodes.

Table S1. Sample ID, GenBank accession number, and identification result for intercepted cerambycids and buprestids.

Sample ID	BOLD Identification Results	Family	Pest?	GenBank accession	Morphology
CA13_15.01	Chrysobothris sp.	Buprestidae	Y	KY357857	
CA13_19.01	Buprestis sp.	Buprestidae	Y	KY357844	
CA13_25.03	Buprestis sp.	Buprestidae	Y	KY357845	
CA14_1.01	Belionota prasina	Buprestidae	Y	KY357832	Y
CA14_31.01	Chrysobothris sp.	Buprestidae	Y	KY357858	
CA14_38.01	Phaenops cyanea	Buprestidae	Y	KY357861	
CA14_4.01	Chrysobothris igniventris	Buprestidae	N	KY357854	
CA14_40.01	Buprestis haemorrhoidalis	Buprestidae	Y	KY357838	
CA14_6.01	Buprestis sp.	Buprestidae	Y	KY357843	
CA15_12.01	Buprestis novemmaculata	Buprestidae	N	KY357837	
CA15_18.01	Inconclusive	Buprestidae	?	KY357859	
CA15_19.01	Inconclusive	Buprestidae	?	KY357835	
FL14_2.01	Inconclusive	Buprestidae	?	KY357860	
MI12_2.01	Chrysobothris igniventris	Buprestidae	N	KY357856	
MI13_14.01	Chrysobothris igniventris	Buprestidae	N	KY357855	
MI13_19.01	Buprestis dalmatina	Buprestidae	Y	KY357877	
MI13_33.01	Belionota sp.	Buprestidae	Y	KY357833	
MI13_33.02	Belionota sp.	Buprestidae	Y	KY357834	
MI14_9.01	Phaenops cyanea	Buprestidae	Y	KY357862	Y
TX13_124.01	Inconclusive	Buprestidae	?	KY357850	
TX13_6.01	Inconclusive	Buprestidae	?	KY357847	
TX13_71.02	Inconclusive	Buprestidae	?	KY357839	
TX13_71.03	Inconclusive	Buprestidae	?	KY357840	
TX13_71.04	Inconclusive	Buprestidae	?	KY357841	
TX13_73.01	Inconclusive	Buprestidae	?	KY357849	
TX13_86.01	Inconclusive	Buprestidae	?	KY357851	
TX13_95.01	Inconclusive	Buprestidae	?	KY357846	
TX14_10.01	Inconclusive	Buprestidae	?	KY357852	
TX14_45.01	Inconclusive	Buprestidae	?	KY357836	
TX15_10.01	Inconclusive	Buprestidae	?	KY357842	
TX15_20.01	Inconclusive	Buprestidae	?	KY357848	
TX15_9.03	Inconclusive	Buprestidae	?	KY357879	
WA13_11.01	Inconclusive	Buprestidae	?	KY357853	
WA13_7.01r	Inconclusive	Buprestidae	?	KY357878	
WA14_14.01	Inconclusive	Buprestidae	?	KY357831	
CA12_1.01	Xylotrechus magnicollis	Cerambycidae	Y	KY357542	Y
CA12_2.01r	Acalolepta sp.	Cerambycidae	N	KY357645	Y

CA12_3.01	Monochamus alternatus	Cerambycidae	Y	KY357735	Y
CA12_3.02r	Stromatium longicorne	Cerambycidae	Y	KY357579	Y
CA12_4.01	Arhopalus sp.	Cerambycidae	N	KY357667	
CA12_5.01	Anoplophora glabripennis	Cerambycidae	Y	KY357652	Y
CA13_1.01	Monochamus galloprovincialis	Cerambycidae	Y	KY357728	
CA13_12.01r	Arhopalus syriacus	Cerambycidae	Y	KY357827	Y
CA13_12.02r	Arhopalus syriacus	Cerambycidae	Y	KY357828	Y
CA13_13.01	Trichoferus sp.	Cerambycidae	Y	KY357609	
CA13_16.03	Inconclusive	Cerambycidae	?	KY357526	
CA13_18.01	Monochamus sp.	Cerambycidae	Y	KY357696	Y
CA13_18.02	Monochamus sutor	Cerambycidae	Y	KY357695	Y
CA13_18.04r	Monochamus sutor	Cerambycidae	Y	KY357694	Y
CA13_18.05r	Monochamus galloprovincialis	Cerambycidae	Y	KY357704	Y
CA13_18.06	Monochamus sutor	Cerambycidae	Y	KY357697	
CA13_18.07	Monochamus urussovi	Cerambycidae	Y	KY357705	
CA13_2.01	Monochamus alternatus	Cerambycidae	Y	KY357739	Y
CA13_20.01	Asemum sp.	Cerambycidae	Y	KY357692	
CA13_20.02	Asemum sp.	Cerambycidae	Y	KY357690	
CA13_23.01	Trichoferus campestris	Cerambycidae	Y	KY357583	Y
CA13_23.02	Trichoferus campestris	Cerambycidae	Y	KY357586	Y
CA13_23.03	Trichoferus campestris	Cerambycidae	Y	KY357585	Y
CA13_23.04	Trichoferus campestris	Cerambycidae	Y	KY357587	
CA13_24.01	Arhopalus sp.	Cerambycidae	N	KY357741	
CA13_26.01	Inconclusive	Cerambycidae	?	KY357826	
CA13_27.01	Xylotrechus buqueti	Cerambycidae	N	KY357555	Y
CA13_28.01	Inconclusive	Cerambycidae	?	KY357876	
CA13_29.01	Arhopalus sp.	Cerambycidae	N	KY357665	
CA13_29.02	Arhopalus sp.	Cerambycidae	N	KY357664	
CA13_31.01	Chlorophorus sp.	Cerambycidae	Y	KY357538	Y
CA13_35.01	Arhopalus sp.	Cerambycidae	N	KY357671	
CA13_36.01	Acanthocinus aedilis	Cerambycidae	N	KY357617	Y
CA13_41.01	Inconclusive	Cerambycidae	?	KY357545	
CA13_42.01	Xystrocera globosa	Cerambycidae	Y	KY357523	Y
CA13_42.02r	Asemum sp.	Cerambycidae	Y	KY357693	
CA13_44.01	Trichoferus campestris	Cerambycidae	Y	KY357597	
CA13_44.02	Trichoferus campestris	Cerambycidae	Y	KY357599	
CA13_44.03	Trichoferus campestris	Cerambycidae	Y	KY357600	
CA13_44.04	Trichoferus campestris	Cerambycidae	Y	KY357588	
CA13_6.01	Anoplophora glabripennis	Cerambycidae	Y	KY357651	Y
CA13_7.01	Pogonocherus perroudi	Cerambycidae	Y	KY357527	Y
CA13_7.03	Pogonocherus perroudi	Cerambycidae	Y	KY357525	Y

CA13_8.01	Anoplophora glabripennis	Cerambycidae	Y	KY357649	
CA13_9.01	Anoplophora glabripennis	Cerambycidae	Y	KY357648	Y
CA14_10.01	Arhopalus rusticus	Cerambycidae	N	KY357658	
CA14_11.01	Xylotrechus rufilius	Cerambycidae	N	KY357540	Y
CA14_12.01	Trichoferus campestris	Cerambycidae	Y	KY357590	
CA14_15.03	Arhopalus sp.	Cerambycidae	N	KY357743	
CA14_17.01	Inconclusive	Cerambycidae	?	KY357528	
CA14_18.01	Arhopalus ferus	Cerambycidae	N	KY357757	
CA14_22.01	Callidium violaceum	Cerambycidae	N	KY357532	
CA14_22.02	Callidium violaceum	Cerambycidae	N	KY357533	
CA14_22.03	Callidium violaceum	Cerambycidae	N	KY357534	
CA14_22.04	Callidium violaceum	Cerambycidae	N	KY357536	
CA14_24.01	Trichoferus campestris	Cerambycidae	Y	KY357595	
CA14_27.01	Anoplophora glabripennis	Cerambycidae	Y	KY357650	
CA14_27.02	Inconclusive	Cerambycidae	?	KY357830	
CA14_29.01	Inconclusive	Cerambycidae	?	KY357524	
CA14_3.01	Acanthocinus griseus	Cerambycidae	Y	KY357619	Y
CA14_3.02	Trichoferus campestris	Cerambycidae	Y	KY357593	
CA14_32.01	Monochamus galloprovincialis	Cerambycidae	Y	KY357726	
CA14_36.01	Inconclusive	Cerambycidae	?	KY357824	
CA14_36.02	Inconclusive	Cerambycidae	?	KY357829	
CA14_36.03	Inconclusive	Cerambycidae	?	KY357825	
CA14_37.01	Trichoferus campestris	Cerambycidae	Y	KY357604	
CA14_41.01	Arhopalus sp.	Cerambycidae	N	KY357679	
CA14_9.01	Phoracantha recurva	Cerambycidae	Y	KY357655	Y
CA14_9.02	Phoracantha recurva	Cerambycidae	Y	KY357653	Y
CA15_01.02	Arhopalus sp.	Cerambycidae	N	KY357670	
CA15_10.01	Inconclusive	Cerambycidae	?	KY357550	
CA15_14.01	Monochamus galloprovincialis	Cerambycidae	Y	KY357730	
CA15_17.01	Arhopalus sp.	Cerambycidae	N	KY357684	
CA15_17.02	Arhopalus sp.	Cerambycidae	N	KY357659	
CA15_21.01	Arhopalus sp.	Cerambycidae	N	KY357661	
CA15_8.01	Arhopalus sp.	Cerambycidae	N	KY357681	
FL13_1.01	Inconclusive	Cerambycidae	?	KY357537	
FL14_1.01	Monochamus sp.	Cerambycidae	Y	KY357706	
MI12_1.01	Monochamus galloprovincialis	Cerambycidae	Y	KY357729	
MI12_1.02	Monochamus galloprovincialis	Cerambycidae	Y	KY357720	
MI12_3.01	Monochamus galloprovincialis	Cerambycidae	Y	KY357721	
MI12_4.01	Arhopalus sp.	Cerambycidae	N	KY357675	
MI12_4.02	Arhopalus sp.	Cerambycidae	N	KY357674	
MI12_5.01	Callidium violaceum	Cerambycidae	N	KY357529	

MI12_5.02	Callidium violaceum	Cerambycidae	N	KY357530	
MI12_6.01	Inconclusive	Cerambycidae	?	KY357627	
MI12_6.02	Inconclusive	Cerambycidae	?	KY357628	
MI12_7.01	Monochamus galloprovincialis	Cerambycidae	Y	KY357733	
MI12_8.01	Anastrangalia dubia	Cerambycidae	N	KY357754	
MI13_1.01	Xylotrechus rusticus	Cerambycidae	Y	KY357561	Y
MI13_10.01	Arhopalus sp.	Cerambycidae	N	KY357668	
MI13_10.02	Arhopalus sp.	Cerambycidae	N	KY357676	
MI13_11.01	Arhopalus sp.	Cerambycidae	N	KY357677	
MI13_13.01	Monochamus sp.	Cerambycidae	Y	KY357702	
MI13_15.01	Trichoferus sp.	Cerambycidae	Y	KY357608	
MI13_16.01	Arhopalus rusticus	Cerambycidae	N	KY357660	
MI13_17.01	Arhopalus sp.	Cerambycidae	N	KY357663	
MI13_18.01	Arhopalus sp.	Cerambycidae	N	KY357748	
MI13_18.02	Arhopalus unicolor	Cerambycidae	N	KY357746	
MI13_18.03	Arhopalus sp.	Cerambycidae	N	KY357745	
MI13_2.01	Arhopalus rusticus	Cerambycidae	N	KY357682	
MI13_21.01	Arhopalus sp.	Cerambycidae	N	KY357669	
MI13_23.01	Inconclusive	Cerambycidae	?	KY357522	
MI13_23.02	Callidium violaceum	Cerambycidae	N	KY357535	
MI13_27.01	Tetropium sp.	Cerambycidae	Y	KY357636	
MI13_27.02	Tetropium sp.	Cerambycidae	Y	KY357635	Y
MI13_28.01	Molorchus minor	Cerambycidae	N	KY357580	
MI13_3.02	Arhopalus sp.	Cerambycidae	N	KY357673	
MI13_30.01	Monochamus galloprovincialis	Cerambycidae	Y	KY357722	
MI13_31.02	Arhopalus sp.	Cerambycidae	N	KY357672	
MI13_32.01	Tetropium castaneum	Cerambycidae	Y	KY357633	
MI13_4.01	Inconclusive	Cerambycidae	?	KY357631	
MI13_4.02	Inconclusive	Cerambycidae	?	KY357630	
MI13_4.03	Inconclusive	Cerambycidae	?	KY357629	
MI13_5.01	Xylotrechus rusticus	Cerambycidae	Y	KY357558	Y
MI13_5.02	Xylotrechus rusticus	Cerambycidae	Y	KY357557	Y
MI13_6.01	Monochamus galloprovincialis	Cerambycidae	Y	KY357727	Y
MI13_7.01	Tetropium castaneum	Cerambycidae	Y	KY357632	
MI14_10.01r	Trichoferus campestris	Cerambycidae	Y	KY357584	
MI14_12.01	Tetropium sp.	Cerambycidae	Y	KY357642	
MI14_12.02	Tetropium sp.	Cerambycidae	Y	KY357640	
MI14_12.05	Tetropium sp.	Cerambycidae	Y	KY357643	
MI14_12.06	Tetropium sp.	Cerambycidae	Y	KY357639	
MI14_12.08	Tetropium sp.	Cerambycidae	Y	KY357634	
MI14_13.01	Monochamus sartor	Cerambycidae	Y	KY357703	Y

MI14_14.01	Arhopalus rusticus	Cerambycidae	N	KY357666	
MI14_18.01	Anastrangalia sp.	Cerambycidae	Y	KY357755	
MI14_18.02	Anastrangalia sp.	Cerambycidae	Y	KY357753	
MI14_18.03	Anastrangalia sp.	Cerambycidae	Y	KY357756	
MI14_18.04	Anastrangalia sp.	Cerambycidae	Y	KY357752	
MI14_21.01	Arhopalus sp.	Cerambycidae	N	KY357678	
MI14_22.01	Inconclusive	Cerambycidae	?	KY357689	
MI14_3.01	Xylotrechus rusticus	Cerambycidae	Y	KY357556	Y
MI14_4.01	Anastrangalia sp.	Cerambycidae	Y	KY357751	
MI14_4.02r	Anastrangalia sp.	Cerambycidae	Y	KY357750	
MI14_5.01	Tetropium castaneum	Cerambycidae	Y	KY357622	Y
MI14_5.03r	Tetropium castaneum	Cerambycidae	Y	KY357638	Y
MI14_5.07	Tetropium fuscum	Cerambycidae	Y	KY357624	Y
MI14_5.08	Tetropium fuscum	Cerambycidae	Y	KY357623	Y
MI14_5.09r	Tetropium fuscum	Cerambycidae	Y	KY357626	Y
MI14_5.10	Tetropium castaneum	Cerambycidae	Y	KY357874	Y
MI14_5.12	Tetropium sp.	Cerambycidae	Y	KY357641	
MI14_8.01	Inconclusive	Cerambycidae	?	KY357541	
MI14_8.02	Inconclusive	Cerambycidae	?	KY357546	
MI14_8.03	Inconclusive	Cerambycidae	?	KY357547	
MI14_8.04	Inconclusive	Cerambycidae	?	KY357544	
MI14_8.05	Inconclusive	Cerambycidae	?	KY357543	
MI14_8.06	Xylotrechus rufilius	Cerambycidae	N	KY357549	Y
MI14_8.07	Inconclusive	Cerambycidae	?	KY357539	
MI14_8.08	Inconclusive	Cerambycidae	?	KY357548	
TX12_1.01	Inconclusive	Cerambycidae	?	KY357875	
TX12_10.01b	Tetropium sp.	Cerambycidae	Y	KY357637	
TX12_11.01	Xylotrechus rusticus	Cerambycidae	Y	KY357562	
TX12_14.01b	Monochamus sp.	Cerambycidae	Y	KY357709	
TX12_15.01	Monochamus sp.	Cerambycidae	Y	KY357712	
TX12_16.01	Monochamus sp.	Cerambycidae	Y	KY357714	
TX12_19.01	Acanthocinus griseus	Cerambycidae	N	KY357620	
TX12_2.01	Monochamus galloprovincialis	Cerambycidae	Y	KY357723	
TX12_20.01	Acanthocinus griseus	Cerambycidae	N	KY357621	
TX12_21.01	Monochamus urussovi	Cerambycidae	Y	KY357719	
TX12_22.01	Monochamus galloprovincialis	Cerambycidae	Y	KY357724	Y
TX12_23.01	Monochamus urussovi	Cerambycidae	Y	KY357715	
TX12_24.01	Monochamus galloprovincialis	Cerambycidae	Y	KY357725	
TX12_25.01	Monochamus sp.	Cerambycidae	Y	KY357718	
TX12_26.01	Monochamus urussovi	Cerambycidae	Y	KY357713	
TX12_27.01	Xylotrechus rusticus	Cerambycidae	Y	KY357559	

TX12_28.01r	Monochamus urussovi	Cerambycidae	Y	KY357710	Y
TX12_29.01	Monochamus sp.	Cerambycidae	Y	KY357717	
TX12_6.01	Arhopalus sp.	Cerambycidae	N	KY357763	
TX12_8.01	Monochamus urussovi	Cerambycidae	Y	KY367700	
TX12_9.01	Monochamus sp.	Cerambycidae	Y	KY357708	Y
TX13_1.01r	Monochamus sartor	Cerambycidae	Y	KY357711	Y
TX13_10.01r	Batocera lineolata	Cerambycidae	Y	KY357614	
TX13_101.01	Arhopalus productus	Cerambycidae	N	KY357769	Y
TX13_102.01	Arhopalus sp.	Cerambycidae	N	KY357791	
TX13_106.01	Arhopalus sp.	Cerambycidae	N	KY357762	
TX13_107.01r	Arhopalus sp.	Cerambycidae	N	KY357781	Y
TX13_108.01	Arhopalus sp.	Cerambycidae	N	KY357774	
TX13_109.01	Arhopalus sp.	Cerambycidae	N	KY357798	Y
TX13_11.01	Arhopalus sp.	Cerambycidae	N	KY357809	
TX13_110.01	Arhopalus productus	Cerambycidae	N	KY357770	Y
TX13_111.01	Arhopalus sp.	Cerambycidae	N	KY357803	
TX13_112.01	Xylotrechus sp.	Cerambycidae	Y	KY357566	
TX13_114.01	Arhopalus sp.	Cerambycidae	N	KY357800	Y
TX13_115.01	Arhopalus sp.	Cerambycidae	N	KY357807	Y
TX13_116.01	Arhopalus sp.	Cerambycidae	N	KY357797	Y
TX13_117.01	Arhopalus sp.	Cerambycidae	N	KY357812	Y
TX13_119.01	Phoracantha recurva	Cerambycidae	Y	KY357654	
TX13_12.0	Trichoferus sp.	Cerambycidae	Y	KY357607	
TX13_120.01	Inconclusive	Cerambycidae	?	KY357611	
TX13_121.01	Monochamus clamator	Cerambycidae	Y	KY357734	Y
TX13_13.01	Arhopalus sp.	Cerambycidae	N	KY357758	
TX13_14.01	Inconclusive	Cerambycidae	?	KY357685	
TX13_15.01	Xylotrechus sp.	Cerambycidae	Y	KY357564	
TX13_15.02	Xylotrechus sp.	Cerambycidae	Y	KY357565	
TX13_17.01	Arhopalus sp.	Cerambycidae	N	KY357810	
TX13_19.01	Arhopalus sp.	Cerambycidae	N	KY357777	
TX13_2.01	Saperda perforata	Cerambycidae	N	KY357644	Y
TX13_20.01	Arhopalus sp.	Cerambycidae	N	KY357806	
TX13_22.01	Arhopalus sp.	Cerambycidae	N	KY357811	
TX13_23.01	Arhopalus sp.	Cerambycidae	N	KY357805	
TX13_25.01	Arhopalus sp.	Cerambycidae	N	KY357815	
TX13_26.01	Arhopalus sp.	Cerambycidae	N	KY357808	
TX13_27.01r	Arhopalus montanus	Cerambycidae	N	KY357817	Y
TX13_29.01	Arhopalus sp.	Cerambycidae	N	KY357816	Y
TX13_3.01	Monochamus sp.	Cerambycidae	Y	KY357698	
TX13_30.01r	Xylotrechus sagittatus	Cerambycidae	Y	KY357571	Y

TX13_31.01	Arhopalus productus	Cerambycidae	N	KY357764	Y
TX13_34.01	Xylotrechus rusticus	Cerambycidae	Y	KY357560	Y
TX13_35.02	Tetropium sp.	Cerambycidae	Y	KY357625	
TX13_36.02	Monochamus sp.	Cerambycidae	Y	KY357699	
TX13_4.01	Inconclusive	Cerambycidae	?	KY357686	
TX13_40.01	Arhopalus sp.	Cerambycidae	N	KY357775	
TX13_41.01	Arhopalus sp.	Cerambycidae	N	KY357820	
TX13_43.01	Arhopalus sp.	Cerambycidae	N	KY357768	
TX13_45.01	Monochamus urussovi	Cerambycidae	Y	KY357707	
TX13_46.01r	Arhopalus rusticus	Cerambycidae	N	KY357662	
TX13_47.01	Xylotrechus sagittatus	Cerambycidae	N	KY357563	Y
TX13_48.01r	Arhopalus rusticus	Cerambycidae	N	KY357683	Y
TX13_5.01	Inconclusive	Cerambycidae	?	KY357616	
TX13_50.01	Xylotrechus sp.	Cerambycidae	Y	KY357569	
TX13_51.01	Xylotrechus sp.	Cerambycidae	Y	KY357567	
TX13_54.01	Arhopalus sp.	Cerambycidae	N	KY357786	
TX13_55.01	Monochamus sp.	Cerambycidae	Y	KY357716	
TX13_57.01	Pseudastylopsis sp.	Cerambycidae	N	KY357610	Y
TX13_58.01	Arhopalus sp.	Cerambycidae	N	KY357793	
TX13_59.01r	Arhopalus montanus	Cerambycidae	N	KY357802	Y
TX13_60.01	Arhopalus sp.	Cerambycidae	N	KY357778	
TX13_61.01	Arhopalus sp.	Cerambycidae	N	KY357767	
TX13_62.01	Arhopalus sp.	Cerambycidae	N	KY357821	
TX13_66.01	Arhopalus sp.	Cerambycidae	N	KY357801	
TX13_67.01	Inconclusive	Cerambycidae	?	KY357615	
TX13_68.01	Monochamus sp.	Cerambycidae	Y	KY357701	
TX13_7.01	Inconclusive	Cerambycidae	?	KY357613	
TX13_70.01	Monochamus galloprovincialis	Cerambycidae	Y	KY357731	
TX13_75.01	Xylotrechus sp.	Cerambycidae	Y	KY357570	
TX13_75.02	Xylotrechus sp.	Cerambycidae	Y	KY357568	
TX13_76.01	Arhopalus sp.	Cerambycidae	N	KY357779	
TX13_77.01r	Arhopalus montanus	Cerambycidae	N	KY357790	Y
TX13_79.01r	Arhopalus montanus	Cerambycidae	N	KY357804	Y
TX13_8.01	Monochamus galloprovincialis	Cerambycidae	Y	KY357732	Y
TX13_82.01r	Arhopalus sp.	Cerambycidae	N	KY357795	Y
TX13_83.01	Arhopalus unicolor	Cerambycidae	N	KY357742	
TX13_85.01r	Arhopalus montanus	Cerambycidae	N	KY357796	Y
TX13_89.01	Arhopalus productus	Cerambycidae	N	KY357760	Y
TX13_93.01	Arhopalus sp.	Cerambycidae	N	KY357783	
TX13_94.01b	Arhopalus sp.	Cerambycidae	N	KY357785	Y
TX13_96.01	Inconclusive	Cerambycidae	?	KY357687	

TX13_97.01	Arhopalus sp.	Cerambycidae	N	KY357794	
TX14_12.01	Arhopalus sp.	Cerambycidae	N	KY357799	
TX14_15.01	Arhopalus sp.	Cerambycidae	N	KY357780	
TX14_16.01	Xylotrechus sp.	Cerambycidae	Y	KY357572	
TX14_21.01	Arhopalus sp.	Cerambycidae	N	KY357787	
TX14_21.02	Arhopalus sp.	Cerambycidae	N	KY357784	
TX14_23.01	Inconclusive	Cerambycidae	?	KY357573	
TX14_24.01	Inconclusive	Cerambycidae	?	KY357823	
TX14_27.01	Arhopalus sp.	Cerambycidae	N	KY357813	
TX14_30.01	Arhopalus sp.	Cerambycidae	N	KY357818	
TX14_30.02	Arhopalus sp.	Cerambycidae	N	KY357814	
TX14_32.01	Arhopalus sp.	Cerambycidae	N	KY357782	
TX14_33.01	Inconclusive	Cerambycidae	?	KY357575	
TX14_36.01	Arhopalus sp.	Cerambycidae	N	KY357788	
TX14_37.01	Arhopalus sp.	Cerambycidae	N	KY357789	
TX14_39.01	Inconclusive	Cerambycidae	?	KY357688	
TX14_4.01	Arhopalus sp.	Cerambycidae	N	KY357771	
TX14_40.01	Inconclusive	Cerambycidae	?	KY357656	
TX14_40.02	Inconclusive	Cerambycidae	?	KY357657	
TX14_43.01	Inconclusive	Cerambycidae	?	KY357574	
TX14_7.01	Arhopalus sp.	Cerambycidae	N	KY357776	
TX14_9.01	Arhopalus sp.	Cerambycidae	N	KY357765	
TX15_11.01	Inconclusive	Cerambycidae	?	KY357576	
TX15_11.02	Inconclusive	Cerambycidae	?	KY357577	
TX15_11.03	Inconclusive	Cerambycidae	?	KY357578	
TX15_12.01	Arhopalus sp.	Cerambycidae	N	KY357759	
TX15_17.01	Arhopalus sp.	Cerambycidae	N	KY357822	
TX15_18.01	Arhopalus sp.	Cerambycidae	N	KY357772	
TX15_19.02	Arhopalus sp.	Cerambycidae	N	KY357773	
TX15_3.01	Arhopalus sp.	Cerambycidae	N	KY357819	
TX15_5.01	Arhopalus sp.	Cerambycidae	N	KY357792	
TX15_6.01	Arhopalus sp.	Cerambycidae	N	KY357766	
WA12_10.02	Inconclusive	Cerambycidae	?	KY357553	
WA12_19.01	Trichoferus campestris	Cerambycidae	Y	KY357594	
WA12_20.01r	Inconclusive	Cerambycidae	?	KY357612	
WA12_20.02	Hylotrupes sp.	Cerambycidae	N	KY357581	
WA12_21.06	Arhopalus sp.	Cerambycidae	N	KY357761	
WA12_22.01	Asemum sp.	Cerambycidae	Y	KY357691	
WA12_24.01	Trichoferus campestris	Cerambycidae	Y	KY357589	
WA12_25.01r	Anoplophora glabripennis	Cerambycidae	Y	KY357647	
WA13_10.01r	Xylotrechus smei	Cerambycidae	N	KY357552	Y

WA13_10.02	Monochamus alternatus	Cerambycidae	Y	KY357736	
WA13_10.03	Inconclusive	Cerambycidae	?	KY357551	
WA13_12.01	Arhopalus sp.	Cerambycidae	N	KY357744	
WA13_13.03r	Arhopalus rusticus	Cerambycidae	N	KY357680	Y
WA13_14.01r	Monochamus alternatus	Cerambycidae	Y	KY357737	Y
WA13_16.01	Inconclusive	Cerambycidae	?	KY357554	
WA13_18.01r	Monochamus alternatus	Cerambycidae	Y	KY357740	Y
WA13_2.01	Inconclusive	Cerambycidae	?	KY357646	
WA13_26.02	Trichoferus campestris	Cerambycidae	Y	KY357602	Y
WA13_26.03	Trichoferus campestris	Cerambycidae	Y	KY357603	Y
WA13_29.03	Trichoferus campestris	Cerambycidae	Y	KY357596	
WA13_30.01	Monochamus alternatus	Cerambycidae	Y	KY357738	
WA13_31.02	Acanthocinus griseus	Cerambycidae	Y	KY357618	Y
WA13_5.01	Trichoferus campestris	Cerambycidae	Y	KY357591	Y
WA14_17.01	Trichoferus campestris	Cerambycidae	Y	KY357598	
WA14_18.01	Trichoferus campestris	Cerambycidae	Y	KY357592	
WA14_18.02	Trichoferus campestris	Cerambycidae	Y	KY357582	
WA14_19.01	Callidium violaceum	Cerambycidae	N	KY357531	
WA14_2.01	Arhopalus sp.	Cerambycidae	N	KY357747	
WA14_2.02	Arhopalus sp.	Cerambycidae	N	KY357749	
WA15_1.01	Trichoferus campestris	Cerambycidae	Y	KY357601	
WA15_1.02	Trichoferus campestris	Cerambycidae	Y	KY357605	
WA15_1.03	Trichoferus campestris	Cerambycidae	Y	KY357606	
MI14-005-05	Tetropium castaneum	Cerambycidae	Y	N/A	Y
TX13-038-01	Derolus sp.	Cerambycidae	N	N/A	Y
TX13-072-01	Arhopalus montanus	Cerambycidae	N	N/A	Y
WA12-021-05	Chlorophorus diadema	Cerambycidae	Y	N/A	Y
WA12-021-01	Chlorophorus diadema	Cerambycidae	Y	N/A	Y
WA12-021-07	Chlorophorus diadema	Cerambycidae	Y	N/A	Y
WA12-021-08	Chlorophorus diadema	Cerambycidae	Y	N/A	Y
WA12-021-09	Chlorophorus diadema	Cerambycidae	Y	N/A	Y
WA13-019-01	Arhopalus rusticus	Cerambycidae	Y	N/A	Y
WA13-023-02	Monochamus alternatus	Cerambycidae	Y	N/A	Y
WA13-026-01	Trichoferus campestris	Cerambycidae	Y	N/A	Y
WA12-027-01	Ceresium sp.	Cerambycidae	Y	N/A	Y

Table S2. Sample ID, GenBank accession number, and BOLD results for submitted insects identified as neither Cerambycidae nor Buprestidae.

Sample ID	BOLD Identification Results	Family	Order	GenBank accession
MI12_6.03r	Wroughtonia dentator	Braconidae	Hymenoptera	KY357863
MI13_8.01	Rhimphoctona sp.	Ichneumonidae	Hymenoptera	KY357864
MI14_1.01r	Rhimphoctona sp.	Ichneumonidae	Hymenoptera	KY357865
TX12_18.01	Zopherus sp.	Zopheridae	Coleoptera	KY357866
TX13_18.01	Zopherus sp.	Zopheridae	Coleoptera	KY357867
TX14_28.01	Inconclusive	?	Hymenoptera	KY357868
TX14_46.01	Inconclusive	?	Coleoptera	KY357869
WA12_3.02	Tremex fuscicornis	Siricidae	Hymenoptera	KY357870
WA12_7.01	Inconclusive	Siricidae	Hymenoptera	KY357871
WA14_12.01	Serropalpus barbatus	Melandryidae	Coleoptera	KY357872
WA14_12.02	Serropalpus barbatus	Melandryidae	Coleoptera	KY357873

Table S3. Origin of overseas shipments and associated SWPM for two groups identified as *Arhopalus* by morphology and/or DNA barcodes.

Sample ID	Commodity Origin	Interception Port
**Group I**		
TX13_41.01_Arhopalus_sp.	Mexico	Laredo TX
TX13_62.01_Arhopalus_sp.	Mexico	Laredo TX
TX13_23.01_Arhopalus_sp.	Mexico	Laredo TX
TX13_116.01_Arhopalus_sp.	Mexico	Laredo TX
TX13_79.01r_Arhopalus_montanus	Mexico	Laredo TX
TX15_17.01_Arhopalus_sp.	Mexico	Laredo TX
TX15_3.01_Arhopalus_sp.	Mexico	Laredo TX
TX13_97.01_Arhopalus_sp.	Mexico	Laredo TX
TX13_85.01r_Arhopalus_montanus	Mexico	Laredo TX
TX13_82.01r_Arhopalus_sp.	Mexico	Laredo TX
TX15_5.01_Arhopalus_sp.	Mexico	Laredo TX
TX13_58.01_Arhopalus_sp.	Mexico	Laredo TX
TX13_117.01_Arhopalus_sp.	Mexico	Laredo TX
TX13_26.01_Arhopalus_sp.	Mexico	Laredo TX
TX14_30.02_Arhopalus_sp.	Mexico	Laredo TX
TX13_25.01_Arhopalus_sp.	Mexico	Laredo TX
TX13_20.01_Arhopalus_sp.	Mexico	Laredo TX
TX14_27.01_Arhoplaus_sp.	Mexico	Laredo TX
TX13_111.01_Arhopalus_sp.	Mexico	Laredo TX
TX14_30.01_Arhopalus_sp.	Mexico	Laredo TX
TX13_27.01r_Arhopalus_montanus	Mexico	Laredo TX
TX13_17.01_Arhopalus_sp.	Mexico	Laredo TX
TX13_115.01_Arhopalus_sp.	Mexico	Laredo TX
TX13_29.01_Arhopalus_sp.	Mexico	Laredo TX
TX13_11.01_Arhopalus_sp.	Mexico	Laredo TX
TX13_22.01_Arhopalus_sp.	Mexico	Laredo TX
TX13_109.01_Arhopalus_sp.	Mexico	Laredo TX
TX13_102.01_Arhopalus_sp.	Mexico	Laredo TX
TX13_114.01_Arhoplaus_sp.	Mexico	Laredo TX
TX13_66.01_Arhopalus_sp.	Mexico	Laredo TX
TX13_59.01r_Arhopalus_montanus	Mexico	Laredo TX
TX14_12.01_Arhopalus_sp.	Mexico	Laredo TX
TX13_54.01_Arhopalus_sp.	Mexico	Laredo TX
TX13_94.01b_Arhopalus_sp.	Mexico	Laredo TX

TX14_21.02_Arhopalus_sp.	Mexico	Laredo TX
TX14_21.01_Arhopalus_sp.	Mexico	Laredo TX
TX14_37.01_Arhopalus_sp.	Mexico	Laredo TX
TX14_36.01_Arhoplaus_sp.	Mexico	Laredo TX
TX13_77.01r_Arhopalus_montanus	Mexico	Laredo TX
TX13_93.01_Arhopalus_sp.	Mexico	Laredo TX
TX13_40.01_Arhopalus_sp.	Mexico	Laredo TX
TX13_108.01_Arhopalus_sp.	Mexico	Laredo TX
TX14_7.01_Arhopalus_sp.	Mexico	Laredo TX
TX15_19.02_Arhopalus_sp.	Mexico	Laredo TX
TX15_18.01_Arhopalus_sp.	Mexico	Laredo TX
TX13_110.01_Arhopalus_productus	Mexico	Laredo TX
TX14_4.01_Arhopalus_sp.	Mexico	Laredo TX
TX13_101.01_Arhopalus_sp.	Mexico	Laredo TX
TX13_43.01_Arhopalus_sp.	Mexico	Laredo TX
TX13_61.01_Arhopalus_sp.	Mexico	Laredo TX
WA12_21.05_Arhopalus_sp.	China	Seattle Sea WA
TX15_6.01_Arhopalus_sp.	Mexico	Laredo TX
TX13_106.01_Arhopalus_sp.	Mexico	Laredo TX
TX14_9.01_Arhopalus_sp.	Mexico	Laredo TX
TX13_31.01_Arhopalus_productus	Mexico	Laredo TX
TX12_6.01_Arhopalus_sp.	Mexico	Laredo TX
Tx13_89.01_Arhopalus_productus	Mexico	Laredo TX
TX14_32.01_Arhopalus_sp.	Mexico	Laredo TX
TX13_107.01r_Arhopalus_sp.	Mexico	Laredo TX
TX13_60.01_Arhoplaus_sp.	Mexico	Laredo TX
TX13_76.01_Arhoplaus_sp.	Mexico	Laredo TX
TX14_15.01_Arhopalus_sp.	Mexico	Laredo TX
TX13_19.01_Arhopalus_sp.	Mexico	Laredo TX
TX15_12.01_Arhopalus_sp.	Mexico	Laredo TX
TX13_13.01_Arhopalus_sp.	Mexico	Laredo TX
CA13_12.02r_Arhopalus_syriacus	Turkey	Long beach CA
CA13_12.01r_Arhopalus_syriacus	Turkey	Long beach CA
CA13_26.01_Inconclusive	Turkey	Long beach CA
CA14_36.02_Inconclusive	Turkey	Long beach CA
CA14_27.02_Inconclusive	China	Long beach CA
CA14_36.01_Inconclusive	Turkey	Long beach CA
CA14_36.03_Inconclusive	Turkey	Long beach CA
TX14_24.01_Inconclusive	South Africa	Houston Sea TX

CA14_18.01_Arhopalus_ferus	Spain	Long beach Ca
WA14_2.02_Arhopalus_sp.	China	Seattle Sea WA
MI13_18.02_Arhopalus_unicolor	China	Romulus MI
CA14_15.03_Arhopalus_sp.	China	Long beach CA
WA14_2.01_Arhopalus_sp.	China	Seattle Sea WA
MI13_18.03_Arhopalus_sp.	China	Romulus MI
WA13_12.01_Arhopalus_sp.	China	Seattle Sea WA
TX13_83.01_Arhopalus_unicolor	China	Houston Sea WA
MI13_18.01_Arhopalus_sp.	China	Romulus MI
CA13_24.01_Arhopalus_sp.	China	Long beach CA
**Group II**		
MI12_4.01_Arhopalus_sp.	Czech Republic	Romulus MI
MI12_4.02_Arhopalus_sp.	Czech Republic	Romulus MI
MI13_3.02_Arhopalus_sp.	Turkey	Romulus MI
CA13_35.01_Arhopalus_sp.	Turkey	Long beach CA
MI13_2.01_Arhopalus_rusticus	Germany	Romulus MI
CA15_17.01_Arhopalus_sp.	Ukraine	Long beach CA
TX13_48.01r_Arhopalus_sp.	Ukraine	Houston TX
CA15_8.01_Arhoplaus_sp.	Ukraine	Long beach CA
CA15_21.01_Arhoplaus_sp.	Ukraine	Long beach CA
CA15_1.02_Arhopalus_sp.	Ukraine	Long beach CA
MI13_10.02_Arhopalus_sp.	Germany	Romulus MI
CA12_4.01_Arhopalus_sp.	France	Long beach CA
MI13_11.01_Arhoplaus_sp.	Turkey	Romulus MI
MI14_21.01_Arhopalus_sp.	Turkey	Romulus MI
CA14_41.01_Arhopalus_sp.	Ukraine	Long beach CA
WA13_13.03r_Arhopalus_sp.	Turkey	Seattle Sea WA
MI14_14.01_Arhopalus_sp.	Czech Republic	Romulus MI
CA13_29.01_Arhopalus_sp.	Italy	Long beach CA
CA13_29.02_Arhoplaus_sp.	Italy	Long beach CA
MI13_17.01_Arhopalus_sp.	Turkey	Romulus MI
TX13_46.01r_Arhoplaus_rusticus	Ukraine	Houston Sea TX
MI13_31.02_Arhopalus_sp.	Czech Republic	Romulus MI
MI13_16.01_Arhopalus_rusticus	Czech Republic	Romulus MI
MI13_10.01_Arhopalus_sp.	Germany	Romulus MI
MI13_21.01_Arhopalus_sp.	United Kingdom	Romulus MI
CA15_17.02_Arhopalus_sp.	Ukraine	Long beach CA
CA14_10.01_Arhopalus_rusticus	United Arab Emirates	Long beach CA

TX12_1.01_Inconclusive	South Korea	Houston Sea TX
